# Occupational lifting and risk of hypertension, stratified by use of anti-hypertensives and age - a cross-sectional and prospective cohort study

**DOI:** 10.1186/s12889-021-10651-w

**Published:** 2021-04-14

**Authors:** Mette Korshøj, Harald Hannerz, Ruth Frikke-Schmidt, Jacob L. Marott, Peter Schnohr, Els Clays, Andreas Holtermann

**Affiliations:** 1 National Research Centre for the Working Environment, Lersø Parkallé 105, 2100 Copenhagen, Denmark; 2 Department of Occupational and Social Medicine, Holbæk Hospital, a part of Copenhagen University Hospital, Gl. Ringstedvej 4B, 4300 Holbæk, Denmark; 3 Department of Clinical Biochemistry, Copenhagen University Hospital, Inge Lehmanns Vej 5, 2100 Copenhagen, Denmark; 4 Copenhagen General Population Study, Herlev-Gentofte Hospital, Borgmester Ib Juuls Vej 1, 2730 Herlev, Denmark; 5 Department of Clinical Medicine, Faculty of Health and Medical Sciences, University of Copenhagen, Blegdamsvej 3B, 2200 Copenhagen, Denmark; 6 Copenhagen City Heart Study, Bispebjerg-Frederiksberg Hospital, Nordre Fasanvej 57, 2000 Frederiksberg, Denmark; 7 Department of Public Health and Primary Care, Ghent University, C. Heymanslaan 10, 9000 Ghent, Belgium; 8 Department of Sports Science and Clinical Biomechanics, University of Southern Denmark, Campusvej 55, 5230 Odense, Denmark

**Keywords:** Cardiovascular risk, Blue-collar occupations, Aging workers, Occupational health, Ergonomics

## Abstract

**Background:**

Heavy occupational lifting is prevalent in the general working population and is sparsely reported to associate with hypertension, especially among older and hypertensive workers. We investigated if heavy occupational lifting is associated with hypertension and blood pressure (BP) in both cross-sectional and prospective study designs in the Copenhagen General Population Study, stratified by age, and use of anti-hypertensives.

**Methods:**

Participation was conducted following the declaration of Helsinki and approved by the ethical committee (H-KF-01-144/01). By multivariable logistic and linear regression models, we investigated the association between heavy occupational lifting and hypertension, in a cross-sectional design (*n* = 67,363), using anti-hypertensives or BP ≥140/≥90 mmHg as outcome, and in a prospective design (*n* = 7020) with an above-median change in systolic BP (SBP) from baseline to follow-up and/or a shift from no use to use of anti-hypertensives as outcome, with and without stratification by age and use of anti-hypertensives.

**Results:**

The odds ratio for hypertension was estimated at 0.97 (99% CI: 0.93–1.00) in the cross-sectional analysis, and at 1.08 (99% CI: 0.98–1.19) in the prospective analysis. The difference in SBP among workers with versus without heavy occupational lifting was estimated at − 0.29 mmHg (99% CI -0.82 – 0.25) in the cross-sectional and at 1.02 mmHg (99% CI -0.41 – 2.45) in the prospective analysis. No significant interaction between heavy occupational lifting and age, nor use of anti-hypertensives were shown.

**Conclusions:**

Only the prospective analysis indicated heavy occupational lifting to increase the risk of hypertension. Further research on the association between occupational lifting and hypertension are needed.

**Supplementary Information:**

The online version contains supplementary material available at 10.1186/s12889-021-10651-w.

## Background

Hypertension increase risk for cardiovascular disease [1, 2]. Prevalence of hypertension vary across occupations and may be affected by occupational exposures, such as heavy lifting [3, 4]. Lifting heavy burdens acutely increases the blood pressure (BP) [5], and several hours of lifting can induce future increases in BP [6]. Only a few studies have investigated the associations of occupational lifting and blood pressure [6–8] and thus more knowledge regarding this association is warranted. Previously, one epidemiological study reported weak positive associations between occupational lifting and BP, especially among users of anti-hypertensives [7]. Additionally, did a cross-sectional study show increases in ambulatory blood pressure, both during work, leisure, and sleep time, by exposure to occupational lifting [6]. Leisure-time physical activity (LTPA) and cardiorespiratory fitness, are known to affect the prevalence of hypertension [9–11]. Among occupational groups exposed to occupational lifting and high levels of occupational physical activity (OPA), a high level of cardiorespiratory fitness is suggested to lower the risk for cardiovascular mortality [12] by reducing the strain on the cardiovascular system [13]. Likewise, have differences in ambulatory BP across sub-groups based on combinations of the level of OPA and LTPA been reported [6], which could be explained by the physical activity health paradox [14]. Thus, for future guidelines of rehabilitation and preventive initiatives, investigations of associations between occupational lifting and BP, stratified in subgroups of levels of LTPA, and the use of anti-hypertensives, would be of interest. Furthermore, are rising age and hypertension known to stiffen the arteries, contributing to endothelial damage, which may increase the total peripheral resistance and thereby also BP [15, 16]. Thus, these two factors potentially increase vulnerability to hazardous effects on BP from exposure to occupational lifting. Hence, to verify previous results, and to additionally investigate the moderating effect from LTPA, we proposed this study aiming to explore associations between heavy occupational lifting and hypertension, stratified on the use of anti-hypertensives, LTPA, and age.

## Methods

Data from the Copenhagen General Population Study was analyzed to replicate the previous results based on the Copenhagen City Heart Study [7], as previously described [17]. Baseline data were collected from 2003 to 2015 and holds information on health as well as a large variety of biological, environmental, and lifestyle-related factors from approximately 110,000 study participants aged 20 to 98 years. Additionally, data from the on-going follow-up data collection, started in 2015 and planned to terminate in 2025, were included. The Copenhagen General Population Study was approved by the local ethical committee (H-KF-01-144/01), participation was conducted following the declaration of Helsinki and all study participants signed informed consent to participate.

The null-hypothesis was no association between heavy occupational lifting and the prevalence of hypertension. Investigation of interactions between age, and heavy occupational lifting, as well as between the use of anti-hypertensives and heavy occupational lifting were planned [17]. In addition, sensitivity analysis of the comparison group for level of OPA and cutpoint for definition of hypertension were performed. As supplementary information associations between heavy occupational lifting and the prevalence of hypertension were reported in groups with combined levels of OPA and LTPA.

### Inclusion criteria

For the cross-sectional analysis, study participants were included by having data on BP, level of OPA (including heavy lifting), use of anti-hypertensives, and being aged ≤70 years old. For the prospective analysis, study participants were included by being normotensive at baseline, having data on the level of OPA at baseline, and data on BP and the use of anti-hypertensives at baseline and follow-up.

### Assessment of exposure

Level of OPA was obtained by use of the question: “Please describe your level of OPA within the past year” with the following response categories:“1) predominantly sedentary; 2) sitting or standing, some walking; 3) walking, some handling of material; 4) heavy manual work”. By answering 3 or 4, an additional question regarding heavy occupational lifting; “Do you lift heavy burdens?” with the response categories: “1) yes; 2) no”, was applied. Study participants were classified as exposed to heavy occupational lifting by answering “yes” to this question, by answering “no” study participants were assigned to the reference group. The stability of exposure was evaluated by Cohen’s kappa by baseline cross-tabulated with follow-up, showing moderate agreement (0.48).

### Assessment of outcome

Primary outcomes were hypertension and SBP (mmHg). Hypertension was classified as using anti-hypertensives and/or SBP ≥ 140 mmHg or DBP ≥ 90 mmHg at baseline examination. For the prospective analysis hypertension was classified as the shift from normotensive not using anti-hypertensives to the use of anti-hypertensives or above median delta value of SBP (follow-up – baseline). Moreover, secondary analyses were conducted to evaluate associations between heavy occupational lifting on pulse pressure (SBP – DBP) and mean arterial pressure (MAP) (2*DBP + SBP)/3). BP was measured on one arm while sitting, after 5 min of rest, using an automated apparatus (BPA3plus, Microlife, Switzerland). The technicians were specially trained and the instruction was the same at both data collection time points.

### Assessment of covariates

Various factors are shown to relate to both occupational workload (including lifting) and BP. Thus, those factors were included in the analyses. The following factors were included as effect modifiers by use of interaction terms: age (categories of < 50; ≥50 years) [18]; the level of LTPA (categories of *mainly sedentary* “you spend most of your leisure-time performing sedentary tasks”; *light physical activity* “you go for a walk, use your bicycle a little or perform an activity for at least 4 hours per week”; *moderate physical activity* “you are an active athlete, for at least 3 hours/week; *strenuous physical activity* “you take part in competitive sports or perform moderate to vigorous activity (MVPA) more than 4 hours/week”) [19]; and use prescription medication for hypertension (anti-hypertensives) (categories of no use of anti-hypertensives; and use of anti-hypertensives). The following factors were included as confounders: sex (male/female) [20]; age (categories of < 40; 40–49; 50–59; 60–70 years) [18]; body mass index (BMI) (categories of < 18.5; 18.5–24.9; 25.0–29.9; ≥30 kg/m^2^) [21]; smoking (categories of nonsmoking; currently smoking) [22]; the level of LTPA (categories of *mainly sedentary* “you spend most of your leisure-time performing sedentary tasks”; *light physical activity* “you go for a walk, use your bicycle a little or perform an activity for at least 4 h/week”; *moderate physical activity* “you are an active athlete, for at least 3 h/week; *strenuous physical activity* “you take part in competitive sports or perform moderate to vigorous activity (MVPA) more than 4 h/week”) [19], mental stress (“are you often feeling nervous or stressed?” yes/no) [23], and length of school education in total years [24].

### Criteria for statistical significance

The overall level of statistical significance, for the cross-sectional and prospective analyses, was set at 0.05. We tested five hypotheses regarding cross-sectional associations and five hypotheses regarding prospective hypotheses. To adjust for multiple comparisons, a Bonferroni correction was applied, thus each of the primary hypotheses was tested at a significance level of 0.01. All secondary analyses were evaluated by the 99% confidence interval (CI) and not statistical significance level, as they were considered exploratory.

### Statistical analyses

All statistical analyses were performed in the statistical software SAS, version 9.4 (SAS Institute, Cary, NC, USA). In the cross-sectional analysis the odds ratio (OR) for being hypertensive, and in the prospective analysis, the odds of becoming a SBP case (defined as a shift from no use to the use of anti-hypertensives and/or an above above-median change in systolic BP (baseline to follow-up)), as a function of heavy occupational lifting, were estimated by use of logistic regression in a generalized estimating equation (GEE) model. Observations from the same person were treated as repeated measurements. A first-order autoregressive correlation structure was assumed. The cross-sectional analysis was controlled for sex, age, BMI, smoking, LTPA, mental stress, school education. No exposure for heavy occupational lifting was the reference. Additionally, the prospective analysis was adjusted for BP at baseline.

Furthermore, the differences in resting SBP across study participants exposed and non-exposed to heavy occupational lifting were analyzed by cross-sectional and prospective (change in mmHg from baseline to follow-up) linear regressions adjusted for sex, age, BMI, smoking, LTPA, mental stress, and school education, and additional BP at baseline in the prospective analysis. To investigate whether age and use of anti-hypertensives moderated the association between heavy occupational lifting and SBP an interaction term was included for each of these variables (exposure*moderating factor). By significant interactions, the linear regressions were applied to groups stratified by age, and the use of anti-hypertensives.

The secondary explorative analyses investigated the effect of heavy occupational lifting on DBP, but not SBP, as previously reported [7]. Also, the linear regressions were repeated for the outcomes of MAP, and PP. Additionally, to investigate the sensitivity of the comparison group, the adjusted primary analyses were repeated in models with a comparison group split by the self-reported categories of OPA, resulting in four instead of two categories. Moreover, to investigate the sensitivity of the definition of hypertension, used in the primary analyses, two alternative definitions of hypertension were applied (SBP ≥160 mmHg or DBP ≥100 mmHg [25]; SBP ≥130 mmHg or DBP ≥80 mmHg [26]). To investigate whether the BP differs between sub-groups defined by levels of OPA, exposure to heavy occupational lifting, LTPA, and use of anti-hypertensives, mean levels of BP across these sub-groups were compared in a generalized linear model adjusted for sex, age, BMI, smoking, mental stress, and school education. Additionally, the odds for being classified as hypertensive, or becoming a SBP case, were investigated in sub-groups defined by the level of LTPA, by use of maximum likelihood and logistic regression, adjusted for sex, age, BMI, smoking, mental stress, school education, and with no exposure for heavy occupational lifting as reference.

## Results

**Fig. 1:**
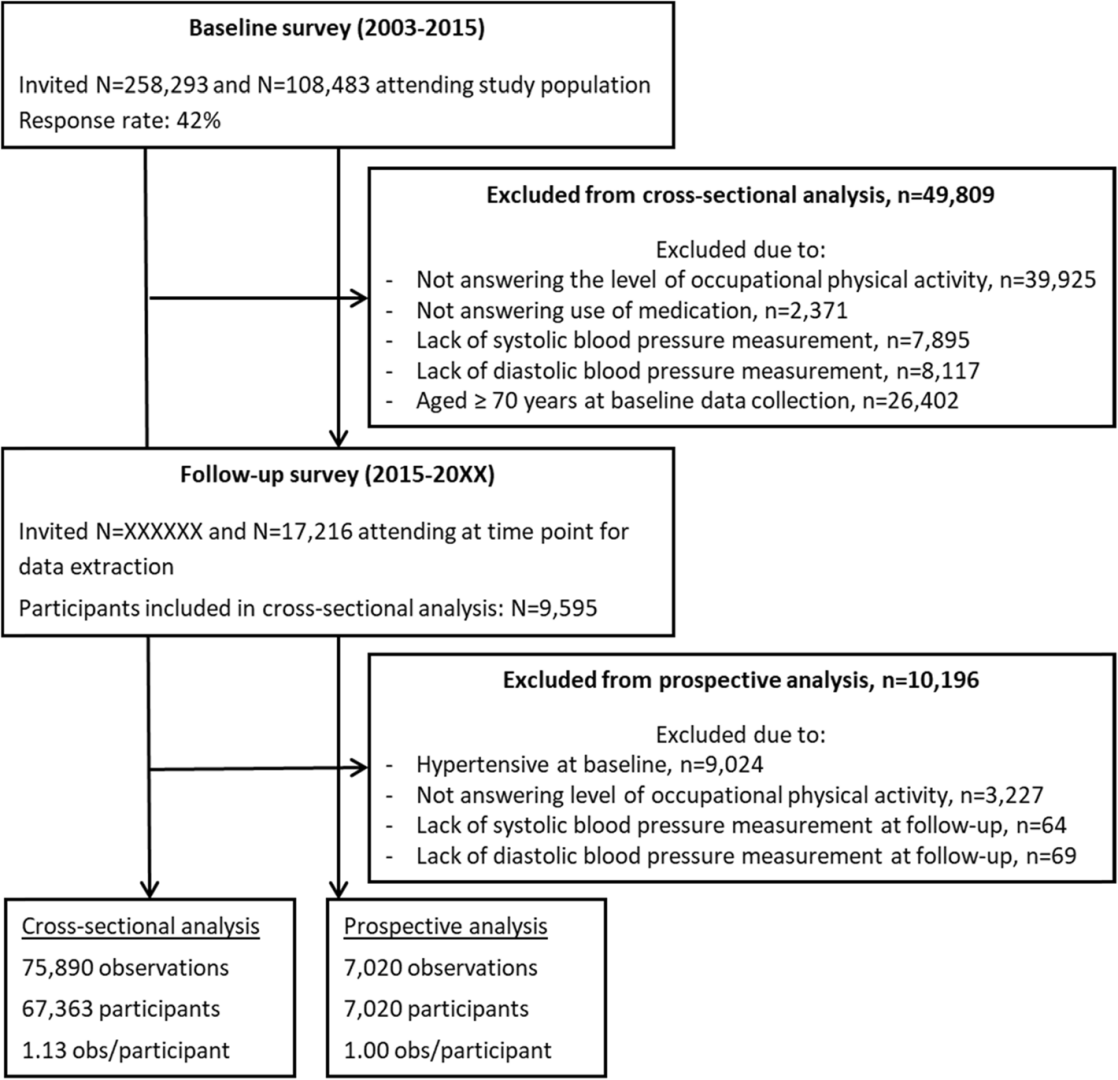
The flow of the observations and study participants in the examination of the Copenhagen General Population Study

From the ongoing follow-up data collection, we had access to responses from 17,216 study participants. Based on the inclusion criteria, the final population for the cross-sectional analysis included 67,363 study participants and 75,890 observations, and the prospective analysis included 7020 observations and study participants (Fig. 1).

### Descriptive information of the study population

Descriptive information on the included observations is presented in Table 1. The included observations in the cross-sectional analysis differed from the excluded observations by being 17.7 years younger (included 51.9 years old, excluded 69.6 years old), having an 11.8 mmHg lower SBP (included 133.2 mmHg, excluded 145.0 mmHg), 19 percentage points (pp) fewer study participants using anti-hypertensives (included 12%, excluded 33%), 9 pp. more were exposed to occupational heavy lifting (included 33%, excluded 24%), 9 pp. more were feeling stressed (included 27%, excluding 18%), and having 1.5 years more school education (included 11.3, excluded 9.8). In the prospective analysis, the included observations differed from the excluded observations by being 9.2 years younger (included 49.2 years old, excluding 58.4 years old), having a 26.0 mmHg lower SBP (included 122.2 mmHg, excluding 148.2 mmHg), 23 pp. fewer study participants using anti-hypertensives (included 0%, excluding 23%), 4 pp. more were exposed to occupational heavy lifting (included 14%, excluded 10%), 6 pp. more were feeling stressed (included 30%, excluding 24%), and having 1.0 years more school education (included 11.2 years, excluding 10.2 years).

**Table 1:** Baseline characteristics of the included 75,890 observations in the cross-sectional analysis and the 7020 study participants in the prospective analysis

	Cross-sectional analysis				Prospective analysis			
	Mean	SD	n (%)	Range	Mean	SD	n (%)	Range
**Age (years)**	51.9	9.5		20.1–69.9	49.2	8.4		20.6–69.8
**Sex (%female)**			41,843 (55.1)				4550 (64.8)	
**BMI (kg/m**^**2**^**)**	25.9	4.3		14.3–91.8	24.5	3.4		15.4–46.9
**Smoking (%current smokers)**			12,666 (16.7)				1366 (19.5)	
**Systolic blood pressure (mmHg)**	133.2	19.5		70.0–240.0	122.2	10.0		71.0–139.0
**Diastolic blood pressure (mmHg)**	79.4	11.5		22.0–190.0	75.9	7.4		30.0–89.0
**Blood pressure ≥ 90/≥140 mmHg**			29,261 (38.6)				–	
**Using anti-hypertensives**			8961 (11.8)				–	
**Being hypertensive (using anti-hypertensives and/or SBP ≥ 140 mmHg or DBP ≥ 90 mmHg at baseline examination)**			32,503 (42.8)				–	
**Being hypertensive (using anti-hypertensives and/or SBP ≥ 130 mmHg or DBP ≥ 80 mmHg at baseline examination)**			50,967 (67.2)				3801 (54.1)	
**Being hypertensive (using anti-hypertensives and/or SBP ≥ 160 mmHg or DBP ≥ 100 mmHg at baseline examination)**			16,137 (21.3)				–	
**School education (years)**	11.3	1.7		0.0–14.0	11.2	1.6		1.0–14.0
**Occupational physical activity**								
**Predominantly sedentary**			33,397 (44.0)				2884 (41.1)	
**Sitting or standing, some walking**			24,877 (32.8)				2284 (32.5)	
**Walking, some handling of material**			14,961 (19.7)				1635 (23.3)	
**Heavy manual work**			2655 (3.5)				217 (3.1)	
**Occupational heavy lifting (%yes)**			9652 (33.0)				990 (14.1)	
**Leisure-time physical activity**								
**Inactive/light physical active < 2 h/week**			4836 (6.4)				401 (5.7)	
**Light physical active 2–4 h/week**			30,531 (40.2)				2936 (41.8)	
**Light physical active > 4 h/week OR MVPA 2–4 h/week**			34,537 (45.5)				3244 (46.2)	
**MVPA > 4 h/week**			5701 (7.5)				416 (5.9)	
**Mental stress (%often feeling nervous or stressed)**			20,473 (27.1)				2112 (30.2)	

Some of these differences between included and excluded observations in the cross-sectional analysis might be explained by the inclusion criteria of answering the question regarding exposure to OPA and heavy occupational lifting and being aged < 70 years old at the time of data collection combined with the fact that 80% of the excluded study participants were unemployed or retired. Additionally, some of the differences may be explained by the inclusion criteria of being normotensive at baseline combined with the fact that 52% of the study participants at follow-up were hypertensive and 32% of the excluded study participants were unemployed or retired at baseline. Thus, the population included in the analysis was younger and overall healthier than the source population.

### Primary and secondary analysis

The adjusted cross-sectional analysis showed that those exposed to heavy occupational lifting had 3% lower odds of hypertension than the non-exposed (Table 2). The adjusted prospective analysis showed an 8% higher risk for being a SBP case by exposure to heavy occupational lifting (Table 2). Likewise showed the secondary analysis among those exposed to heavy occupational lifting a 2% higher odds of becoming a DBP case (Table 2). Linear regressions were performed to investigate the differences in mmHg of SBP, and secondary DBP, PP, and MAP, between study participants exposed and non-exposed to heavy occupational lifting. No associations were seen in either the cross-sectional analysis or prospective analysis, neither in the fully adjusted or crude models (Table 3 and Table S1, S2, and S3).

**Table 2:** Adjusted odds ratios of being hypertensive (in the cross-sectional model) and for becoming a SBP or DBP case, defined as an above-median delta value of BP at follow-up – BP at baseline and/or a shift from no use to use of anti-hypertensives (in the prospective model) as a function of self-rated exposure to heavy occupational lifting, with and without stratification on age at baseline (≥ vs. < 50 years). No exposure to heavy occupational lifting was the reference category. [OR = Odds ratio; CI=Confidence interval]. Significant OR are highlighted in bold

	Occupational lifting	Cross-sectional model			Prospective model				
					Systolic blood pressure case			Diastolic blood pressure case	
		n	OR’	99% CI	n	OR	99% CI	OR	99% CI
**All**^**a**^	Yes	9591	0.97	0.93–1.00	990	1.08	0.98–1.19	1.02	0.93–1.12
	No	65,596	1.00	–	6030	1.00	–	1.00	–
**Age < 50 years**^**a**^	Yes	4048	0.96	0.91–1.02	566	1.05	0.93–1.19	1.01	0.89–1.14
	No	26,391	1.00	–	3251	1.00	–	1.00	–
**Age ≥ 50 years**^**a**^	Yes	5540	0.97	0.93–1.01	424	1.11	0.95–1.29	1.04	0.90–1.21
	No	39,184	1.00	–	2777	1.00	–	1.00	–

^a^adjusted for sex, age, BMI, smoking, LTPA, mental stress, and school education, and additionally SBP at baseline in the prospective analysis

**Table 3:** Adjusted linear regressions on systolic blood pressure (SBP) as a function of heavy occupational lifting without and with stratification by age, and use of anti-hypertensives. Significant associations are highlighted in bold

	Occupational lifting	Cross-sectional model Difference in systolic blood pressure			Prospective model Difference in delta systolic blood pressure		
		n	Β^**a**^ (mmHg)	99% CI	n	Β^**a**^ (mmHg)	99% CI
**All**^**a**^	Yes	9591	−0.29	−0.82 – 0.25	990	1.02	−0.41 – 2.45
	No	65,596	0.00	–	6030	0.00	–
**Age < 50 years**^**a**^	Yes	4048	− 0.12	− 0.86 – 0.62	566	1.40	− 0.47 – 3.26
	No	26,391	0.00	–	3251	0.00	–
**Age ≥ 50 years**^**a**^	Yes	5540	−0.23	− 0.98 – 0.52	424	0.45	−1.75 – 2.66
	No	39,184	0.00	–	2777	0.00	–
**NOT using anti-hypertensives**^**a**^	Yes	8442	−0.21	−0.77 – 0.35	930	1.20	−0.27 – 2.67
	No	57,826	0.00	–	5769	0.00	–
**USING anti-hypertensives**^**a**^	Yes	1149	−0.55	−2.17 – 1.06	60	−2.01	−7.77 – 3.75
	No	7770	0.00	–	261	0.00	–

^a^adjusted for sex, age, BMI, smoking, LTPA, mental stress, and school education, and additionally SBP at baseline in the prospective analysis

In the cross-sectional analysis, significant interactions (*p* < 0.0001) were found between heavy occupational lifting and age, and the use of anti-hypertensives, thus stratified analyses were performed. The age-stratified analysis showed insignificant odds for becoming a SBP or DBP case by exposure to heavy occupational lifting, being somewhat higher for the older than the younger study participants (Table 2). Yet, the age-stratified linear regression did not show any associations between heavy occupational lifting and difference in SBP at baseline or delta SBP at follow-up (Table 3). The linear regressions stratified by use of anti-hypertensives did not show any associations neither in the cross-sectional nor the prospective analysis, except for a minimally higher pulse pressure (0.54 mmHg, 99% CI 0.13–0.94) and MAP (0.49 mmHg, 99% CI 0.12–0.86) among study participants not using anti-hypertensives (Table 3).

In summary, the cross-sectional analysis showed no clear associations. The prospective analysis showed a trend of heavy occupational lifting to be associated with an increased risk of hypertension. Higher numerical OR for becoming a SBP or DBP case when exposed to heavy occupational lifting were seen among workers aged ≥50 years.

### Sensitivity analysis: influence of comparison group on study findings

The study participants were stratified by their self-reported level of OPA to test the sensitivity to the choice of the comparison group. The cross-sectional analysis showed exposure to OPA primarily including sitting or standing and some walking to associate to minimally higher SBP compared to predominantly sedentary work. The prospective model showed positive associations between the level of OPA and SBP, pointing towards exposure for heavy occupational lifting to give the greatest rise in SBP compared to the reference group performing predominantly sedentary work (Table S4).

Additionally, the sensitivity to cut-point for the definition of hypertension was tested by the OR for being hypertensive at both lower (SBP ≥130 mmHg/DBP ≥80 mmHg) and higher (SBP ≥160 mmHg/DBP ≥100 mmHg) cut-points, than those applied in the primary analysis (SBP ≥140 mmHg/DBP ≥90 mmHg). Significantly decreased prevalence of hypertension (4 and 3%) by exposure to heavy occupational lifting was seen in the adjusted models for the two highest cut-points (Table S5).

Hence, level of OPA without exposure to heavy occupational lifting impacted SBP, but the OPA group involving heavy occupational lifting still showed the highest SBP values. The sensitivity of hypertension cutpoint followed the logical notion of the higher the cutpoint used, the lower the prevalence of hypertension seen.

### Supplementary analysis: influence of leisure time physical activity on study findings

The interaction between level of LTPA and heavy occupational lifting was significant and thus logistic and linear regressions stratified by level of LTPA were performed. The logistic regression showed no associations, except a reduced odds for hypertension by exposure to heavy occupational lifting among those performing light physical activity 2–4 h/week (Table S8). The linear regression showed no associations except among those being inactive or performing light physical activity during leisure time where exposure to heavy occupational lifting was associated with a decrease in SBP (− 5.92 mmHg, 99% CI -11.59 - -0.25 mmHg) (Table S9).

The supplementary analysis of differences in mean baseline BP across groups stratified by level of OPA and LTPA showed that the higher the level of LTPA the lower the SBP (*p* value < 0.05) across all levels of OPA (Table S6). Differences in mean baseline BP across sub-populations stratified by level of OPA, LTPA, and use of anti-hypertensives showed an overall trend (*p* value ≤0.03, except for those stating their OPA to be “Moderate and strenuous - with occupational lifting” and not using anti-hypertensives) of the higher the level of LTPA the lower mean BP across all levels of OPA, among those not using anti-hypertensives. However, the level of LTPA did not seem to affect BP among users of anti-hypertensives (Table S6). The differences in follow-up mean SBP and DBP, stratified by level of OPA and LTPA, showed that the higher the level of LTPA the lower the mean SBP and DBP were among study participants reporting exposure to light OPA (predominantly sitting or standing, including some walking) (*p* value ≤0.03) (Table S7). No differences in SBP and DBP were seen across the remaining OPA and LTPA groups. Differences in mean BP at follow-up, across subpopulations stratified by level of OPA, LTPA, and use of anti-hypertensives, showed no differences in BP, except among study participants reporting to be exposed to light OPA (predominantly sitting or standing, including some walking) and not using anti-hypertensives, where a higher level of LTPA related to a lower mean SBP and DBP across all levels of OPA (*p* value ≤0.03) (Table S7).

Thus, overall level of LTPA did not have a major impact on the associations between heavy occupational lifting and BP and hypertension among those not using antihypertensives, with a few exceptions. Nonetheless, a clear pattern of the more LTPA, the lower the BP was seen, but only among those not using antihypertensives.

## Discussion

This study contributes to the knowledge on risk for hypertension from heavy occupational lifting by its aim to verify previous findings [7], and to perform further analysis accounting for the moderating effects of LTPA. Thus, this study explored associations between heavy occupational lifting and hypertension in the Copenhagen General Population Study. The adjusted cross-sectional analysis indicated a 3% lower risk of hypertension by exposure to heavy occupational lifting (Table 2), which was supported by the adjusted linear associations between heavy occupational lifting and SBP (mmHg) indicating a negative association (− 0.29 mmHg, 99% CI -0.82 – 0.25 mmHg, Table 3). These associations could be explained by the cross-sectional design of this analysis, meaning that this result may be owed to either i) exposure to heavy occupational lifting to lower the risk for hypertension or ii) those exposed to heavy occupational lifting being less frequently hypertensive than those not. Within occupational medicine studies, results are assumed to be prone to healthy worker selection bias, implicating less healthy workers migration into occupational groups less exposed to heavy occupational lifting or other strenuous activities [27]. Thus, one could speculate that the acute peaks in BP, while performing lifting tasks [5], may give rise to angina [28], among the workers with poor cardiovascular health. Hence, workers experiencing angina or such would be more likely to migrate into less strenuous occupational groups.

On the contrary, the adjusted prospective analysis indicated an 8% higher risk for being a SBP case by exposure to heavy occupational lifting (Table 2), while the linear regressions showed an increase in SBP (1.59 mmHg, 99% CI 0.05–3.13 mmHg, Table S4) among those exposed to heavy occupational lifting. Opposite to the BP effects from resistance training during LTPA [29, 30], these results indicate heavy occupational lifting to have hazardous effects on BP, as previously indicated [7]. The background for increased risk for hypertension by exposure to heavy occupational lifting, may lie within the repeated acute peaks in BP during lifting tasks [5]. The acute BP peaks occurs due to the occlusion of the vessels induced by static muscle activity leading to increases in total peripheral resistance [15]. During heavy occupational lifting these BP peaks are repeated, both during the 7–9 h workday, as well as during the 5-day work-week. Thereby the recovery between BP peaks may be insufficient [14, 31], and could give rise to the increased BP both during working hours as well as across the 24 h BP [6]. However, this higher risk of being a SBP case by exposure to heavy occupational lifting is not reflected in the linear regressions, showing no associations between heavy occupational lifting and SBP, DBP, PP, and MAP (Table 3 and Table S1, S2, and S3). Thus, these findings ought to be interpreted with care.

The age-stratified prospective analysis showed exposure to heavy occupational lifting to increase the risk for being a SBP case, similar to a previous study [7]. The risk was 11% higher among workers aged ≥50 years and 5% higher among workers aged < 50 years (Table 2). Older workers are likely to have been occupationally active throughout a longer time than younger workers, and therefore might the effect of the occupational exposures be more pronounced. Furthermore, a higher strain from heavy occupational lifting will be expected among older than younger workers, due to the combination of age-related declines in aerobic capacity [13] and arterial compliance [15, 16], giving rise to a greater increase in BP and thus a potentially higher risk of hypertension [6].

The adjusted OR for being hypertensive by exposure to heavy occupational lifting, stratified by level of LTPA, showed a minimal numerical tendency of increasing level of LTPA to increase risk for hypertension and for being a SBP case (Table S8). However, these weak associations do not support the common assumption of a beneficial effect of LTPA to decrease the risk of hypertension [19, 25, 26, 32]. Thus, the total volume of physical activity (combining LTPA, OPA and heavy occupational lifting), may result in overstrain, and cardiovascular damage, rather than optimized cardiovascular health. This notion is supported by previous findings among both veteran athletes [33], and workers having combinations of high OPA and high LTPA [34, 35]. Nonetheless, the beneficial effect from LTPA on BP was reflected in the baseline mean BP, showing increased LTPA to relate to a lower BP, regardless of OPA level (Table S6). However, at follow-up the overall mean BP did not show any effect of LTPA, independently of the level of OPA (Table S7). Thus, the tendencies of BP effects from combinations of LTPA and OPA, only seen in the cross-sectional analysis, could be explained by the stratification on level of LTPA and OPA were made by baseline values, also in the prospective analysis, and therefore may the effect have vanished during the 10-year follow-up. Conclusively, as causal effects cannot be drawn from the cross-sectional analysis, these presented results do not indicate the level of LTPA to affect BP across OPA strata.

The analysis stratified by the use of anti-hypertensives did not show results indicating users of anti-hypertensives to be especially vulnerable to rises in BP when exposed to heavy occupational lifting, as previously shown [7]. Yet, users of anti-hypertensives did not seem to have beneficial lowering BP effects by increasing the level of LTPA, in the cross-sectional analysis (Table S6). Previously, greater or similar beneficial effects on BP from LTPA have been seen among hypertensives compared to normotensives [19, 36]. However, these previous studies did not take OPA or heavy occupational lifting into consideration. Thus, future investigations on the effects of LTPA on BP among working-age adults should account for the level of OPA. Hence, to develop recommendations for the prevention of hypertension more knowledge is needed, especially targeted to vulnerable groups of older workers. Also, further investigations are needed to uncover the potential for prevention of progression of hypertension among users of anti-hypertensives, as the level of LTPA does not seem to have the assumed BP-lowering effect [19, 36].

### Methodological considerations

The population included in the cross-sectional analysis were younger, less hypertensive, better educated, and more frequently exposed to heavy occupational lifting than those excluded from the analysis. For the prospective analysis, the differences between those in- and excluded from analysis were similar to the cross-sectional analysis. Besides the inclusion criteria of answering the question regarding exposure to OPA and heavy occupational lifting and being aged < 70 years at baseline, these differences may be explained by the frequency of excluded study participants being retired or unemployed (80% in the cross-sectional data and 32% in the prospective data). Moreover, the inclusion criteria for the prospective analysis of being normotensive excluded 52% of the study participants at follow-up. Taken together, these differences between in- and excluded study participants indicate that the population analyzed was overall healthier, but also more frequently exposed to heavy occupational lifting, and thus the results may reflect associations being more conservative than if based on the entire sample of study participants. Also, as the complete follow-up sample is currently being collected, these associations should be repeated for verification in the complete sample.

A limitation of the study is the self-reported exposure measure of heavy occupational lifting, as self-reports of ergonomic work exposures have been found imprecise and at risk of recall [37, 38], and social desirability bias, compared to exposures collected by worn devices as accelerometers [39]. The self-reported exposure to heavy occupational lifting is therefore inadequate for a more detailed description of the frequency and duration of lifting tasks and weight of the lifted burden. Due to the dichotomized response category of the exposure to heavy occupational lifting question (yes/no) the responses might be biased of the individual worker’s perception of what heavy occupational lifting are, as no categories of the weight of the lifted burdens are given as an example. Thus, the evaluated exposure to heavy occupational lifting is assumed to be a quite crude indication of exposure to occupational lifting or not, and further investigation by the use of more detailed and accurate measures of exposure to occupational lifting is therefore warranted. Another limitation is the single measurement of a casual BP, shown to have a lower prognostic value than ambulatory BP or BP monitored during sleep [40, 41]. The main strengths of this study include the limited risk of false-negative classification of hypertension due to the determination of hypertension based on both use of anti-hypertensives and the casual BP, in mmHg, and the high number of randomly selected study participants in the study population.

### Perspectives of the proposed findings

The Eurofound survey states that 33% of the European workforce is exposed to occupational lifting (6^th^ survey in Eurofound). Knowledge of the impact on cardiovascular health from occupational lifting is sparse, and to be able to develop preventive initiatives, vocational rehabilitation and clinical guidelines, investigations of the effect of heavy occupational lifting on precursors of cardiovascular disease should be encouraged. In this paper, the associations indicated workers aged ≥50 years to have an increased risk for hypertension (OR 1.11, 99% CI 0.95–1.29, Table 2), when exposed to heavy occupational lifting. Thus this group holds the potential for prevention of hypertension by minimizing exposure to heavy occupational lifting, e.g. by automatization of manual work tasks or use of assistive devices. Also, when planning vocational rehabilitation among workers aged ≥50 years performing heavy occupational lifting and reporting light to moderate levels of LTPA, the exposure to heavy occupational lifting ought to be reduced and strenuous LTPA ought to be promoted. Further, the lower effect of LTPA on reduction of BP among users of anti-hypertensives (Tables S6 and S7), should be kept in mind. Thus, for initiatives targeting primary prevention of hypertension, these results point towards reduction of the overall exposure of heavy occupational lifting and performance of strenuous LTPA for keeping the BP below hypertension cutpoints. For initiatives targeting secondary prevention of hypertension these results points towards reduction of the overall exposure to heavy occupational lifting, especially among those aged ≥50 years.

Furthermore, future preventive initiatives and clinical guidelines should strive to modify the exposure to heavy occupational lifting, as well as stay informed on the growing knowledge of the effects on BP from the combined LTPA and OPA levels to avoid the risk of cardiovascular overstrain. Thus, to develop preventive initiatives, vocational rehabilitation and clinical guidelines for heavy occupational lifting in relation to risk for hypertension more knowledge is needed.

## Conclusion

No associations between heavy occupational lifting and BP nor hypertension were found in the cross-sectional analysis. The prospective analysis shows a trend towards the exposure of heavy occupational lifting to increase the risk of hypertension, especially among workers aged ≥50 years or reporting light to moderate levels of LTPA.

## Supplementary Information

**Additional file 1 Table S1.** Adjusted linear regressions on diastolic BP (mmHg) as a function of heavy occupational lifting, without and with stratification by age and use of anti-hypertensives. [CI=Confidence interval]. The reference was no exposure to heavy occupational lifting. Significant associations are highlighted in bold.

**Additional file 2: Table S2.** Adjusted linear regressions on pulse pressure (mmHg) as a function of heavy occupational lifting, without and with stratification by age and use of anti-hypertensives. [CI=Confidence interval]. The reference was no exposure to heavy occupational lifting. Significant associations are highlighted in bold.

**Additional file 3: Table S3.** Adjusted linear regressions on mean arterial pressure (mmHg) as a function of heavy occupational lifting, without and with stratification by age and use of anti-hypertensives. [CI=Confidence interval]. The reference was no exposure to heavy occupational lifting. Significant associations are highlighted in bold.

**Additional file 4: Table S4.** Adjusted associations between self-reported heavy occupational lifting and systolic blood pressure (mmHg), stratified by level of occupational physical activity. [β = Difference in mmHg; CI = Confidence interval; Ref. = Reference group].

**Additional file 5: Table S5.** Cross-sectional odd ratios (OR) for being hypertensive by exposure to heavy occupational lifting, when hypertension is defined at different cutpoints. The reference was no exposure to heavy occupational lifting.

**Additional file 6: Table S6.** Adjusted baseline means of SBP and DBP in groups stratified by OPA with or without occupational lifting combined by LTPA and with or without the use of anti-hypertensives.

**Additional file 7: Table S7.**Adjusted mean of SBP and DBP, at follow-up, in groups stratified by OPA with or without occupational lifting combined by LTPA and with or without the use of anti-hypertensives.

**Additional file 8: Table S8.** Adjusted odds ratios of being hypertensive (in the cross-sectional model) and for becoming a SBP or DBP case (defined as an above-median delta value of BP at follow-up – BP at baseline and/or a shift from no use to use of anti-hypertensives (in the prospective model)) as a function of self-rated exposure to heavy occupational lifting, stratified by level of leisure-time physical activity. No exposure to heavy occupational lifting was the reference category. [OR = Odds ratio; CI=Confidence interval]. Significant OR are highlighted in bold.

**Additional file 9: Table S9.** Adjusted linear regressions on systolic blood pressure (SBP) as a function of heavy occupational lifting stratified level of leisure-time physical activity. Significant associations are highlighted in bold.

## Data Availability

Requests for data should be directed and approved by the Steering Committee of the Copenhagen General Population Study, including co-author Ruth Frikke-Schmidt being a steering group member.

## Abbreviations

OPA: Occupational physical activity
LTPA: Leisure-time physical activity
BMI: Body mass index
BP: Blood pressure
SBP: Systolic blood pressure
DBP: Diastolic blood pressure
pp.: Percentage points
GEE: Generalized estimation equation
OR: Odds ratio
CI: Confidence interval
MVPA: Moderate to vigorous physical activity
