# Supplementary material for: Occupational lifting and risk of hypertension, stratified by use of anti-hypertensives and age - a cross-sectional and prospective cohort study
Source: BMC Public Health. 2021 Apr 14;21:721. doi: 10.1186/s12889-021-10651-w (PMC8045338; doi:10.1186/s12889-021-10651-w)
Supplement: Supplementary file 1 — Additional file 1 Table S1. Adjusted linear regressions on diastolic BP (mmHg) as a function of heavy occupational lifting, without and with stratification by age and use of anti-hypertensives. [CI=Confidence interval]. The reference was no exposure to heavy occupational lifting. Significant associations are highlighted in bold. [file 12889_2021_10651_MOESM1_ESM.docx]

**Supplementary table 1**

**Table S1. Adjusted linear regressions on diastolic BP (mmHg) as a function of heavy occupational lifting, without and with stratification by age and use of anti-hypertensives. [CI=Confidence interval]. The reference was no exposure to heavy occupational lifting. Significant associations are highlighted in bold.**

|  | **Occupa-tional lifting** | **Cross-sectional model**  **Difference in diastolic blood pressure** | | | **Prospective model**  **Difference in delta diastolic blood pressure** | | |
| --- | --- | --- | --- | --- | --- | --- | --- |
|  |  | **n** | **Β* (mmHg)** | **99% CI** | **n** | **Β* (mmHg)** | **99% CI** |
| **All*** | Yes | 9,591 | -0.23 | -0.56 – 0.10 | 990 | 0.87 | -0.03 – 1.76 |
|  | No | 65,596 | 0.00 | - | 6,030 | 0.00 | - |
| **Age < 50 years*** | Yes | 4,048 | -0.32 | -0.82 – 0.17 | 566 | 0.76 | -0.46 – 1.99 |
|  | No | 26,391 | 0.00 | - | 3,251 | 0.00 | - |
| **Age ≥ 50 years*** | Yes | 5,540 | -0.17 | -0.61 - 026 | 424 | 1.00 | -0.30 – 2.31 |
|  | No | 39,184 | 0.00 | - | 2,777 | 0.00 | - |
| **NOT using anti-hypertensives*** | Yes | 8,442 | -0.30 | -0.65 – 0.04 | 930 | 0.79 | -0.14 – 1.71 |
|  | No | 57,826 | 0.00 | - | 5,769 | 0.00 | - |
| **USING anti-hypertensives*** | Yes | 1,149 | 0.37 | -0.55 – 1.29 | 60 | 2.06 | -1.73 – 5.85 |
|  | No | 7,770 | 0.00 | - | 261 | 0.00 | - |

* adjusted for sex, age, BMI, smoking, LTPA, mental stress, and school education, and additionally SBP at baseline in the prospective analysis.
