# Supplementary material for: Occupational lifting and risk of hypertension, stratified by use of anti-hypertensives and age - a cross-sectional and prospective cohort study
Source: BMC Public Health. 2021 Apr 14;21:721. doi: 10.1186/s12889-021-10651-w (PMC8045338; doi:10.1186/s12889-021-10651-w)
Supplement: Supplementary file 2 — Additional file 2: Table S2. Adjusted linear regressions on pulse pressure (mmHg) as a function of heavy occupational lifting, without and with stratification by age and use of anti-hypertensives. [CI=Confidence interval]. The reference was no exposure to heavy occupational lifting. Significant associations are highlighted in bold. [file 12889_2021_10651_MOESM2_ESM.docx]

**Supplementary table 2**

**Table S2. Adjusted linear regressions on pulse pressure (mmHg) as a function of heavy occupational lifting, without and with stratification by age and use of anti-hypertensives.** **[CI=Confidence interval]. The reference was no exposure to heavy occupational lifting. Significant associations are highlighted in bold.**

|  | **Occupa-tional lifting** | **Cross-sectional model**  **Difference in pulse pressure** | | | **Prospective model**  **Difference in delta pulse pressure** | | |
| --- | --- | --- | --- | --- | --- | --- | --- |
|  |  | **n** | **Β* (mmHg)** | **99% CI** | **n** | **Β* (mmHg)** | **99% CI** |
| **All*** | Yes | 9,591 | -0.05 | -0.44 – 0.35 | 990 | 0.15 | -1.09 – 1.40 |
|  | No | 65,596 | 0.00 | - | 6,030 | 0.00 | - |
| **Age < 50 years*** | Yes | 4,048 | 0.20 | -0.34 – 0.75 | 566 | 0.63 | -0.89 – 2.16 |
|  | No | 26,391 | 0.00 | - | 3,251 | 0.00 | - |
| **Age ≥ 50 years*** | Yes | 5,540 | -0.04 | -0.60 – 0.52 | 424 | -0.55 | -2.63 – 1.53 |
|  | No | 39,184 | 0.00 | - | 2,777 | 0.00 | - |
| **NOT using anti-hypertensives*** | Yes | 8,442 | 0.54 | 0.13 – 0.94 | 930 | 0.41 | -0.87 – 1.69 |
|  | No | 57,826 | 0.00 | - | 5,769 | 0.00 | - |
| **USING anti-hypertensives*** | Yes | 1,149 | -0.92 | -2.18 – 0.34 | 60 | -4.07 | -9.46 – 1.32 |
|  | No | 7,770 | 0.00 | - | 261 | 0.00 | - |

* adjusted for sex, age, BMI, smoking, LTPA, mental stress, and school education, and additionally SBP at baseline in the prospective analysis.
