# Supplementary material for: Occupational lifting and risk of hypertension, stratified by use of anti-hypertensives and age - a cross-sectional and prospective cohort study
Source: BMC Public Health. 2021 Apr 14;21:721. doi: 10.1186/s12889-021-10651-w (PMC8045338; doi:10.1186/s12889-021-10651-w)
Supplement: Supplementary file 3 — Additional file 3: Table S3. Adjusted linear regressions on mean arterial pressure (mmHg) as a function of heavy occupational lifting, without and with stratification by age and use of anti-hypertensives. [CI=Confidence interval]. The reference was no exposure to heavy occupational lifting. Significant associations are highlighted in bold. [file 12889_2021_10651_MOESM3_ESM.docx]

**Supplementary table 3**

**Table S3. Adjusted linear regressions on mean arterial pressure (mmHg) as a function of heavy occupational lifting, without and with stratification by age and use of anti-hypertensives.** **[CI=Confidence interval]. The reference was no exposure to heavy occupational lifting. Significant associations are highlighted in bold.**

|  | **Occupa-tional lifting** | **Cross-sectional model**  **Difference in mean arterial pressure** | | | **Prospective model**  **Difference in delta mean arterial pressure** | | |
| --- | --- | --- | --- | --- | --- | --- | --- |
|  |  | **N** | **Β* (mmHg)** | **99% CI** | **n** | **Β* (mmHg)** | **99% CI** |
| **All*** | Yes | 9,591 | -0.25 | -0.62 – 0.11 | 990 | 0.92 | -0.01 – 1.85 |
|  | No | 65,596 | 0.00 | - | 6,030 | 0.00 | - |
| **Age < 50 years*** | Yes | 4,048 | -0.25 | -0.78 – 0.28 | 566 | 0.97 | -0.31 – 2.25 |
|  | No | 26,391 | 0.00 | - | 3,251 | 0.00 | - |
| **Age ≥ 50 years*** | Yes | 5,540 | -0.20 | -0.69 – 0.30 | 424 | 0.82 | -0.52 – 2.16 |
|  | No | 39,184 | 0.00 | - | 2,777 | 0.00 | - |
| **NOT using anti-hypertensives*** | Yes | 8,442 | 0.49 | 0.12 – 0.86 | 930 | 0.93 | -0.04 – 1.89 |
|  | No | 57,826 | 0.00 | - | 5,769 | 0.00 | - |
| **USING anti-hypertensives*** | Yes | 1,149 | 0.06 | -0.98 – 1.10 | 60 | 0.70 | -3.06 – 4.47 |
|  | No | 7,770 | 0.00 | - | 261 | 0.00 | - |

* adjusted for sex, age, BMI, smoking, LTPA, mental stress, and school education, and additionally SBP at baseline in the prospective analysis.
