# Supplementary material for: Occupational lifting and risk of hypertension, stratified by use of anti-hypertensives and age - a cross-sectional and prospective cohort study
Source: BMC Public Health. 2021 Apr 14;21:721. doi: 10.1186/s12889-021-10651-w (PMC8045338; doi:10.1186/s12889-021-10651-w)
Supplement: Supplementary file 4 — Additional file 4: Table S4. Adjusted associations between self-reported heavy occupational lifting and systolic blood pressure (mmHg), stratified by level of occupational physical activity. [β = Difference in mmHg; CI = Confidence interval; Ref. = Reference group]. [file 12889_2021_10651_MOESM4_ESM.docx]

**Supplementary table 4**

**Table S4. Adjusted associations between self-reported heavy occupational lifting and systolic blood pressure (mmHg), stratified by level of occupational physical activity. [β = Difference in mmHg; CI = Confidence interval; Ref. = Reference group].**

| **Occupational physical activity** | **Cross-sectional model**  **N = 77,154** | | **Prospective model**  **N = 6,774** | |
| --- | --- | --- | --- | --- |
|  | **β (mmHg)** | **99% CI** | **β (mmHg)** | **99% CI** |
| **Heavy lifting** | -0.11 | -0.69 – 0.46 | 1.59 | 0.05 – 3.13 |
| **Walking, some handling of material or heavy manual work, but no heavy lifting** | -0.10 | -0.68 – 0.48 | 0.61 | -0.91 – 2.13 |
| **Sitting or standing, some walking** | 0.52 | 0.10 – 0.93 | 1.24 | 0.09 – 2.40 |
| **Predominantly sedentary work** | Ref | - | Ref | - |

**The adjusted cross-sectional model includes adjustment for sex, age, BMI, smoking, LTPA, mental stress, and school education. The prospective models are additionally adjusted for BP at baseline.**
