# Supplementary material for: Occupational lifting and risk of hypertension, stratified by use of anti-hypertensives and age - a cross-sectional and prospective cohort study
Source: BMC Public Health. 2021 Apr 14;21:721. doi: 10.1186/s12889-021-10651-w (PMC8045338; doi:10.1186/s12889-021-10651-w)
Supplement: Supplementary file 5 — Additional file 5: Table S5. Cross-sectional odd ratios (OR) for being hypertensive by exposure to heavy occupational lifting, when hypertension is defined at different cutpoints. The reference was no exposure to heavy occupational lifting. [file 12889_2021_10651_MOESM5_ESM.docx]

**Supplementary table 5**

**Table S5. Cross-sectional odd ratios (OR) for being hypertensive by exposure to heavy occupational lifting, when hypertension is defined at different cutpoints. The reference was no exposure to heavy occupational lifting.**

| **Heavy occupational lifting** | **Crude model** | | | | **Adjusted model** | | | |
| --- | --- | --- | --- | --- | --- | --- | --- | --- |
|  | **N** | **OR** | **99% CI** | **P-value** | **N** | **OR** | **99% CI** | **P-value** |
| **Hypertension definition at (160/100)** | 80,152 | 1.02 | 0.99 – 1.05 | 0.12 | 72,873 | 0.96 | 0.92 – 1.00 | 0.01 |
| **Hypertension definition at (140/90)** | 75,887 | 1.08 | 1.05 – 1.11 | <0.0001 | 72,873 | 0.97 | 0.93 – 1.00 | 0.01 |
| **Hypertension definition at (130/80)** | 75,887 | 1.12 | 1.09 – 1.16 | <0.0001 | 72,873 | 0.99 | 0.96 – 1.03 | 0.56 |

**The adjusted cross-sectional model includes adjustment for sex, age, BMI, smoking, LTPA, mental stress, and school education.**
