# Supplementary material for: Occupational lifting and risk of hypertension, stratified by use of anti-hypertensives and age - a cross-sectional and prospective cohort study
Source: BMC Public Health. 2021 Apr 14;21:721. doi: 10.1186/s12889-021-10651-w (PMC8045338; doi:10.1186/s12889-021-10651-w)
Supplement: Supplementary file 6 — Additional file 6: Table S6. Adjusted baseline means of SBP and DBP in groups stratified by OPA with or without occupational lifting combined by LTPA and with or without the use of anti-hypertensives. [file 12889_2021_10651_MOESM6_ESM.docx]

**Supplementary table 6**

**Table S6. Adjusted baseline means of SBP and DBP in groups stratified by OPA with or without occupational lifting combined by LTPA and with or without the use of anti-hypertensives.**

|  |  | **ALL** | | | | **NOT using anti-hypertensives** | | | | **USING anti-hypertensives** | | | |
| --- | --- | --- | --- | --- | --- | --- | --- | --- | --- | --- | --- | --- | --- |
| **OPA** | **LTPA** | **n** | **SBP (mmHg)** | **DBP (mmHg)** | **Difference across level of LTPA, adjusted model SBP/DBP** | **n** | **SBP (mmHg)** | **DBP (mmHg)** | **Difference across level of LTPA, adjusted model SBP/DBP** | **n** | **SBP (mmHg)** | **DBP (mmHg)** | **Difference across level of LTPA, adjusted model SBP/DBP** |
| **Sedentary** | **Sedentary** | 2,399 | 135.7 | 81.6 | <0.0001/<0.0001 | 1,983 | 133.9 | 80.9 | <0.001/  <0.0001 | 416 | 144.2 | 84.7 | 0.45/0.09 |
|  | **Light** | 12,377 | 133.5 | 79.9 |  | 10,794 | 131.9 | 79.2 |  | 1,583 | 144.1 | 85.2 |  |
|  | **Moderate** | 15,550 | 131.3 | 78.4 |  | 14,110 | 130.1 | 77.8 |  | 1,440 | 142.8 | 84.2 |  |
|  | **Strenuous** | 2,808 | 131.0 | 77.7 |  | 2,624 | 130.2 | 77.2 |  | 184 | 142.2 | 85.0 |  |
| **Light** | **Sedentary** | 1,304 | 136.2 | 81.2 | <0.0001/<0.0001 | 1,073 | 134.5 | 80.8 | <0.0001/<0.0001 | 231 | 143.8 | 83.2 | 0.79/0.13 |
|  | **Light** | 10,636 | 135.3 | 80.4 |  | 8,974 | 133.5 | 79.7 |  | 1,662 | 144.9 | 84.3 |  |
|  | **Moderate** | 10,788 | 132.2 | 78.7 |  | 9,601 | 130.8 | 78.0 |  | 1,187 | 143.9 | 83.9 |  |
|  | **Strenuous** | 1,433 | 130.3 | 77.3 |  | 1,327 | 129.4 | 76.8 |  | 106 | 142.3 | 83.9 |  |
| **Moderate - no occupational lifting** | **Sedentary** | 449 | 133.9 | 80.4 | 0.04/0.03 | 387 | 133.0 | 79.8 | 0.02/0.03 | 62 | 139.6 | 84.1 | 0.10/0.50 |
|  | **Light** | 3,587 | 133.9 | 79.9 |  | 3,132 | 132.7 | 79.3 |  | 455 | 142.4 | 83.7 |  |
|  | **Moderate** | 4,057 | 132.0 | 78.7 |  | 3,651 | 130.5 | 78.0 |  | 406 | 145.6 | 84.8 |  |
|  | **Strenuous** | 616 | 131.1 | 78.0 |  | 583 | 130.3 | 77.6 |  | 33 | 145.4 | 83.4 |  |
| **Moderate and strenuous - with occupational lifting** | **Sedentary** | 684 | 137.0 | 81.7 | 0.01/<0.01 | 595 | 135.9 | 81.2 | 0.07/<0.01 | 89 | 143.9 | 85.0 | 0.11/0.69 |
|  | **Light** | 3,931 | 135.5 | 80.9 |  | 3,419 | 134.0 | 80.2 |  | 512 | 145.4 | 85.9 |  |
|  | **Moderate** | 4,141 | 134.0 | 80.0 |  | 3,670 | 132.9 | 79.4 |  | 471 | 142.3 | 84.8 |  |
|  | **Strenuous** | 844 | 133.5 | 79.1 |  | 771 | 132.7 | 78.5 |  | 73 | 142.3 | 85.5 |  |

**The adjusted cross-sectional model includes adjustment for sex, age, BMI, smoking, mental stress, and school education.**
