# Supplementary material for: Occupational lifting and risk of hypertension, stratified by use of anti-hypertensives and age - a cross-sectional and prospective cohort study
Source: BMC Public Health. 2021 Apr 14;21:721. doi: 10.1186/s12889-021-10651-w (PMC8045338; doi:10.1186/s12889-021-10651-w)
Supplement: Supplementary file 7 — Additional file 7: Table S7.Adjusted mean of SBP and DBP, at follow-up, in groups stratified by OPA with or without occupational lifting combined by LTPA and with or without the use of anti-hypertensives. [file 12889_2021_10651_MOESM7_ESM.docx]

**Supplementary table 7**

**Table S7.Adjusted mean of SBP and DBP, at follow-up, in groups stratified by OPA with or without occupational lifting combined by LTPA and with or without the use of anti-hypertensives.**

|  |  | **ALL** | | | | **NOT using anti-hypertensives** | | | | **USING anti-hypertensives** | | | |
| --- | --- | --- | --- | --- | --- | --- | --- | --- | --- | --- | --- | --- | --- |
| **OPA** | **LTPA** | **n** | **SBP (mmHg)** | **DBP (mmHg)** | **Adjusted model SBP/DBP** | **n** | **SBP (mmHg)** | **DBP (mmHg)** | **Adjusted model SBP/DBP** | **n** | **SBP (mmHg)** | **DBP (mmHg)** | **Adjusted model SBP/DBP** |
| **Sedentary** | **Sedentary** | 191 | 127.3 | 75.5 | 0.10/0.53 | 178 | 126.7 | 75.4 | 0.12/0.50 | 61 | 133.2 | 77.7 | 0.48/0.45 |
|  | **Light** | 1,141 | 126.1 | 74.8 |  | 1,093 | 125.9 | 74.6 |  |  |  |  |  |
|  | **Moderate** | 1,334 | 124.9 | 74.6 |  | 1,287 | 124.6 | 74.5 |  | 54 | 133.6 | 78.2 |  |
|  | **Strenuous** | 191 | 125.8 | 75.7 |  | 184 | 125.5 | 75.5 |  |  |  |  |  |
| **Light** | **Sedentary** | 100 | 130.5 | 77.1 | 0.03/0.02 | 97 | 130.7 | 77.4 | 0.01/0.03 | 57 | 134.2 | 77.1 | 0.45/0.44 |
|  | **Light** | 994 | 128.1 | 75.3 |  | 940 | 127.8 | 75.2 |  |  |  |  |  |
|  | **Moderate** | 1,026 | 126.2 | 74.5 |  | 986 | 125.8 | 74.5 |  | 42 | 137.1 | 75.9 |  |
|  | **Strenuous** | 95 | 127.7 | 76.1 |  | 93 | 127.6 | 75.9 |  |  |  |  |  |
| **Moderate - no occupational lifting** | **Sedentary** | 49 | 127.5 | 75.7 | 0.97/0.77 | 47 | 127.1 | 76.2 | 0.87/0.74 | 28 | 136.1 | 78.8 | 0.40/0.71 |
|  | **Light** | 401 | 125.8 | 75.3 |  | 375 | 125.1 | 75.0 |  |  |  |  |  |
|  | **Moderate** | 438 | 125.3 | 74.3 |  | 421 | 124.9 | 74.0 |  | 18 | 134.8 | 79.9 |  |
|  | **Strenuous** | 52 | 125.8 | 74.3 |  | 51 | 125.9 | 74.3 |  |  |  |  |  |
| **Moderate and strenuous - with occupational lifting** | **Sedentary** | 61 | 128.2 | 74.9 | 0.88/0.17 | 56 | 128.3 | 74.6 | 0.91/0.19 | 36 | 133.9 | 79.4 | 0.80/0.66 |
|  | **Light** | 400 | 127.4 | 76.1 |  | 369 | 126.7 | 75.8 |  |  |  |  |  |
|  | **Moderate** | 446 | 128.1 | 76.4 |  | 426 | 127.7 | 76.2 |  | 24 | 136.0 | 79.6 |  |
|  | **Strenuous** | 78 | 127.9 | 75.4 |  | 74 | 127.9 | 75.7 |  |  |  |  |  |

**The adjusted prospective model includes adjustment for sex, age, BMI, smoking, mental stress, school education, and BP at baseline. *OPA categories of “*Moderate – no occupational lifting*” and “*Moderate and strenuous – with occupational lifting*” combined due to low n-values.**
