# Supplementary material for: Occupational lifting and risk of hypertension, stratified by use of anti-hypertensives and age - a cross-sectional and prospective cohort study
Source: BMC Public Health. 2021 Apr 14;21:721. doi: 10.1186/s12889-021-10651-w (PMC8045338; doi:10.1186/s12889-021-10651-w)
Supplement: Supplementary file 8 — Additional file 8: Table S8. Adjusted odds ratios of being hypertensive (in the cross-sectional model) and for becoming a SBP or DBP case (defined as an above-median delta value of BP at follow-up – BP at baseline and/or a shift from no use to use of anti-hypertensives (in the prospective model)) as a function of self-rated exposure to heavy occupational lifting, stratified by level of leisure-time physical activity. No exposure to heavy occupational lifting was the reference category. [OR = Odds ratio; CI=Confidence interval]. Significant OR are highlighted in bold. [file 12889_2021_10651_MOESM8_ESM.docx]

**Supplementary table 8**

**Table S8. Adjusted odds ratios of being hypertensive (in the cross-sectional model) and for becoming a SBP or DBP case (defined as an above-median delta value of BP at follow-up – BP at baseline** **and/or a shift from no use to use of anti-hypertensives (in the prospective model)) as a function of self-rated exposure to heavy occupational lifting, stratified by level of leisure-time physical activity. No exposure to heavy occupational lifting was the reference category. [OR=Odds ratio; CI=Confidence interval]. Significant OR are highlighted in bold.**

|  | **Occupa-tional lifting** | **Cross-sectional model** | | | **Prospective model** | | | | |
| --- | --- | --- | --- | --- | --- | --- | --- | --- | --- |
|  |  |  |  |  | **Systolic blood pressure case** | | | **Diastolic blood pressure case** | |
|  |  | **n** | **OR’** | **99% CI** | **n** | **OR** | **99% CI** | **OR** | **99% CI** |
| **Inactive/light physical active < 2 hours/week#** | Yes | 682 | 0.95 | 0.84 – 1.07 | 61 | 0.89 | 0.59 – 1.32 | 0.87 | 0.58 – 1.30 |
|  | No | 4,128 | 1.00 | - | 340 | 1.00 | - | 1.00 | - |
| **Light physical active 2-4 hours/week#** | Yes | 3,914 | **0.94** | **0.89 – 0.99** | 400 | 1.05 | 0.90 – 1.22 | 1.07 | 0.92 – 1.24 |
|  | No | 26,416 | 1.00 | - | 2,536 | 1.00 | - | 1.00 | - |
| **Moderate to vigorous physical activity 2-4 hours/week#** | Yes | 4,112 | 0.99 | 0.94 – 1.04 | 446 | 1.13 | 0.98 – 1.31 | 1.03 | 0.90 – 1.19 |
|  | No | 30,046 | 1.00 | - | 2,798 | 1.00 | - | 1.00 | - |
| **Moderate to vigorous physical activity >4 hours/week#** | Yes | 832 | 1.02 | 0.91 – 1.14 | 78 | 1.03 | 0.72 – 1.47 | 0.84 | 0.59 – 1.19 |
|  | No | 4,775 | 1.00 | - | 338 | 1.00 | - | 1.00 | - |

# adjusted for sex, age, BMI, smoking, mental stress, and school education, and additionally SBP at baseline in the prospective analysis.
