# Supplementary material for: Occupational lifting and risk of hypertension, stratified by use of anti-hypertensives and age - a cross-sectional and prospective cohort study
Source: BMC Public Health. 2021 Apr 14;21:721. doi: 10.1186/s12889-021-10651-w (PMC8045338; doi:10.1186/s12889-021-10651-w)
Supplement: Supplementary file 9 — Additional file 9: Table S9. Adjusted linear regressions on systolic blood pressure (SBP) as a function of heavy occupational lifting stratified level of leisure-time physical activity. Significant associations are highlighted in bold. [file 12889_2021_10651_MOESM9_ESM.docx]

**Supplementary table 9**

**Table S9.** **Adjusted linear regressions on systolic blood pressure (SBP) as a function of heavy occupational lifting stratified level of leisure-time physical activity.** **Significant associations are highlighted in bold.**

|  | **Occupa-tional lifting** | **Cross-sectional model**  **Difference in systolic blood pressure** | | | **Prospective model**  **Difference in delta systolic blood pressure** | | |
| --- | --- | --- | --- | --- | --- | --- | --- |
|  |  | **n** | **Β* (mmHg)** | **99% CI** | **n** | **Β* (mmHg)** | **99% CI** |
| **Inactive/light physical active < 2 hours/week#** | Yes | 682 | 0.00 | -2.01 – 2.00 | 61 | **-5.92** | **-11.59 - -0.25** |
|  | No | 4,128 | 0.00 | - | 340 | 0.00 | - |
| **Light physical active 2-4 hours/week#** | Yes | 3,914 | -0.41 | -1.26 – 0.45 | 400 | 0.86 | -1.34 – 3.06 |
|  | No | 26,416 | 0.00 | - | 2,536 | 0.00 | - |
| **Moderate to vigorous physical activity 2-4 hours/week#** | Yes | 4,112 | -0.22 | -1.03 – 0.58 | 446 | 1.80 | -0.27 – 3.87 |
|  | No | 30,046 | 0.00 | - | 2,798 | 0.00 | - |
| **Moderate to vigorous physical activity >4 hours/week#** | Yes | 832 | 0.19 | -1.56 – 1.95 | 78 | 2.23 | -3.50 – 7.97 |
|  | No | 4,775 | 0.00 | - | 338 | 0.00 | - |

# adjusted for sex, age, BMI, smoking, mental stress, and school education, and additionally SBP at baseline in the prospective analysis.
